# Preserved implicit mentalizing in schizophrenia despite poor explicit performance: evidence from eye tracking

**DOI:** 10.1038/srep34728

**Published:** 2016-10-05

**Authors:** Paul Roux, Pauline Smith, Christine Passerieux, Franck Ramus

**Affiliations:** 1 Laboratoire de Sciences Cognitives et Psycholinguistique, UMR 8554, CNRS-ENS-EHESS, Institut d’études de la cognition, Ecole Normale Supérieure, PSL Research University, 29 Rue d’Ulm, 75005, Paris, France; 2 Service Universitaire de Psychiatrie d’adultes, Centre Hospitalier de Versailles, 177 rue de Versailles, 78157 Le Chesnay, France; 3 Laboratoire HandiRESP EA4047, Université Versailles Saint-Quentin-en- Yvelines, 2 Avenue de la Source de la Bièvre, 78180 Montigny-le-Bretonneux, France; 4 Fondation Fondamental, Créteil, France

## Abstract

Schizophrenia has been characterized by an impaired mentalizing. It has been suggested that distinguishing implicit from explicit processes is crucial in social cognition, and only the latter might be affected in schizophrenia. Two other questions remain open: (1) Is schizophrenia characterized by an hypo- or hyper attribution of intentions? (2) Is it characterized by a deficit in the attribution of intention or of contingency? To test these three questions, spontaneous mentalizing was tested in 29 individuals with schizophrenia and 29 control subjects using the Frith-Happé animations, while eye movements were recorded. Explicit mentalizing was measured from participants’ verbal descriptions and was contrasted with implicit mentalizing measured through eye tracking. As a group, patients made less accurate and less intentional descriptions of the goal-directed and theory of mind animations. No group differences were found in the attribution of contingency. Eye tracking results revealed that patients and controls showed a similar modulation of eye movements in response to the mental states displayed in the Frith-Happé animations. To conclude, in this paradigm, participants with schizophrenia showed a dissociation between explicit and implicit mentalizing, with a decrease in the explicit attribution of intentions, whereas their eye movements suggested a preserved implicit perception of intentions.

Schizophrenia is characterized by impairments in several domains of social cognition, including Theory of Mind (ToM) or mentalizing^1^,^2^,^3^ and goal or intention attribution^4^,^5^,^6^. Mentalizing deficits have a clear functional importance in schizophrenia, as they are one of the strongest predictors of functional outcome among the other social as well as non-social cognitive domains^7^. Overall, research on social cognition in schizophrenia leaves some questions open, including three that will be our main focus here: (1) Do individuals with schizophrenia show a hypo- or a hyper-mentalizing deficit? (2) Is this deficit characterized by an impairment in the attribution of intention or contingency? (3) Is this deficit situated at an implicit and automatic or an explicit and reflexive level of processing? Hypomentalizing refers to being less able to perceive and infer goals and intentions. In contrast, hypermentalizing would involve over-attributing intentions, including to non-intentional stimuli. Hypermentalizing has been suggested by several authors by the existence of paranoid symptoms in schizophrenia, leading to an excessive attribution of malevolent intentions to others^8^,^9^. This hypothesis has received some experimental evidence at the behavioral^10^,^11^,^12^,^13^ and the neurophysiological levels^14^,^15^,^16^. Nevertheless, replications of this result are inconsistent. Several studies have reported hypomentalizing without hypermentalizing in schizophrenia^17^,^18^,^19^,^20^. In another study, hypermentalizing in schizophrenia was entirely explained by lower verbal intelligence and memory, which was not the case for hypomentalizing^21^. It has also been suggested that hypermentalizing may be related to apophenia, i.e. an increased tendency to perceive connections between unrelated events^22^. However, only one study reported that patients with delusions of persecution over-attributed contingency, whereas no differences were found with control or non-deluded groups for the attribution of intentions^23^. The issue of hypo- vs. hyper-mentalizing in schizophrenia thus remains largely open.

The other important issue is the distinction between implicit and explicit processing, which Frith and Frith (2008) have convincingly argued is of great relevance to the study of social cognition. Implicit mentalizing involves fast, automatic and relatively inflexible routines while explicit mentalizing rather reflects later, reflexive, decision-making and reporting processes^24^,^25^,^26^,^27^. Social cognitive deficits in patients with schizophrenia might arise at low-level and early perceptual stages. Indeed, a whole section of the literature on schizophrenia is devoted to deficits in basic auditory and visual perceptual processes^28^,^29^, including impairments in the visual exploration of static visual scenes^30^. However, social cognitive deficits in schizophrenia might also arise at higher-level cognitive stages of assessing perceptual evidence. Indeed, in 2004, Frith suggested that social cognition in schizophrenia was characterized by a dissociation between an impaired explicit mentalizing and a spared implicit mentalizing. One major concern in this debate is that the tasks that have been used to assess putatively perceptual abilities or implicit mentalizing, often involve off-line, reflexive and explicit judgments, thereby leaving open several interpretations of patients’ poor performance^9^. In a closely related area of social cognition, some studies reported preserved implicit perception of facial^31^,^32^ and vocal emotions^33^ in individuals with schizophrenia whereas their explicit recognition was altered. However, to our knowledge, no study has directly compared implicit and explicit mentalizing in the same group of patients with schizophrenia within the same paradigm.

Among the various paradigms designed to assess mentalizing in schizophrenia, we chose the Frith-Happé animations because it has been validated across a number of studies. Because participants are simply asked to report spontaneously what they have seen, this task does not require to solve a problem, as opposed to others classical ToM paradigms. In these animations, two triangles are moving according to random (R), goal-directed (GD) and ToM scenarios. In the R condition, the triangles drift and bounce independently like billiard balls whereas, in the GD condition, one triangle appears to act intentionally towards another triangle. Finally, in the ToM condition, the triangles seem to interact in a mentalistic manner, with one triangle trying to influence the mental states of the other (for example one triangle tries to trick the second). Individuals with schizophrenia typically provide less intentional and less accurate descriptions of GD and ToM scenarios than control participants^13^,^17^,^18^,^19^,^34^. The first aim of this study was to explore whether there were deficits in the explicit attribution of mental states and/or contingency to Frith-Happé animations in schizophrenia and whether these deficits were in the direction of a hyper- or hypo attribution.

However, it remains unclear whether mentalizing deficits in schizophrenia are primary or whether they are a consequence of other cognitive impairments like executive functioning. In the present study, we chose to investigate the influence of one particular executive function, contextual processing because the deficit in contextual information integration has been considered as a central pathophysiological mechanism of cognitive impairment in schizophrenia^35^,^36^,^37^. Moreover, it has been suggested that ToM and contextual processing deficits may reflect a single underlying cognitive impairment in schizophrenia^38^. Finally, impairments in context processing have been demonstrated to be related to mentalizing deficits in schizophrenia^39^,^40^. These considerations, therefore, suggest the hypothesis that the deficit in explicit mentalizing found in schizophrenia on Frith-Happé animations may entirely explain by the deficit in contextual processing. We tested this hypothesis by running covariance analyses on mentalizing measures drawn from Frith-Happé animation, with contextual control, verbal and performance intelligence as covariates.

This study also tested Frith’s (2004) hypothesis by recording eye movements on Frith-Happé animations to obtain a more implicit measure of mentalizing in schizophrenia. Although Frith-Happé stimuli elicit spontaneous, largely implicit and automatic mentalizing, putting the content of what is viewed into words is an explicit process. It requires an explicit recognition and an off-line verbalization of these mental states. Eyetracking can assess whether participants can focus attention on socially salient aspects of animations: for example, it has been recently shown that the impairment in false belief inference found in schizophrenia on an object displacement paradigm was related to a deficit in visual attention toward gaze orientation^41^. Eye tracking has also been used to assess implicit mentalizing on Frith-Happé animations in healthy participants, and systematic differences in gaze patterns have been demonstrated for R, GD and ToM animations. First, fixation duration has been used as an index of mentalizing as it increased from R to GD to ToM animations^42^. This increase was interpreted as reflecting complex cognitive processing related to the integration of mental states: in the field of scene perception or reading, fixation duration is considered as a sensitive indicator of processing depth, an increase of fixation duration being associated with more complex processing or integration of various types of information^43^,^44^,^45^. Secondly, gaze directed to the triangles were longer from R to GD to ToM, thus suggesting that eye movements were preferentially drawn to social agents^46^. Finally, it has been suggested that eye tracking differences between ToM and GD were explained by idiosyncratic low-level kinematic properties of the Frith–Happé animations, whereas the differences between R on the one hand and GD/ToM, on the other hand, remained significant even after taking into account kinematic confounds^47^. Thus, the eye tracking measures gathered on Frith-Happé animations can be considered as quantitative and implicit markers of intentional interpretations^48^. To our knowledge, no study has yet explored eye movements for Frith-Happé animations in schizophrenia. According to Frith’s hypothesis, although differences in the verbal descriptions of Frith-Happé animations between participants with schizophrenia and controls are expected based on previous research, the patterns of their eye movements are predicted to be equivalent. If this were the case, this would provide evidence for a dissociation between preserved implicit mentalizing and disrupted explicit mentalizing in schizophrenia.

## Methods

### Participants

29 individuals with schizophrenia and 29 control participants were recruited in this study. All participants had normal or corrected-to-normal vision. Exclusion criteria for both groups comprised substances or alcohol dependence within the past 6 months and current or prior history of untreated significant medical illness or of neurological illness. Two licensed psychiatrists confirmed all diagnoses in the schizophrenia group according to the DSM-IV-R criteria for schizophrenia. Patients were stabilized and were recruited from community mental health centers and outpatient clinics in the Versailles area. At the time of testing, all participants with schizophrenia were taking antipsychotics. The control group was screened for current or past psychiatric illness and participants were excluded if they met criteria for any axis I disorder of the DSM-IV-TR. The experiment was approved by the local medical ethics committee (*Comité de Protection des Personnes Ile-de-France XI*) and was carried out in accordance with the approved guidelines. All participants received a complete description of the study in oral and written form. Written informed consent was obtained from each participant.

### Neuropsychological and psychopathological assessments

General intelligence was estimated by using four WAIS-III subtests: Vocabulary and Similarities for verbal intelligence; Picture Completion and Matrices for non-verbal intelligence. Executive functioning was evaluated by using a contextual cognitive control paradigm^49^ that has previously demonstrated a contextual processing deficit in patients with schizophrenia^35^. The task is fully described in Supplementary Information 1. We rated the severity of schizophrenic symptoms in all patients with the Positive and Negative Syndrome Scale (PANSS)^50^.

### Frith-Happé Paradigm

We used the full-size version of the 12 Frith-Happé animation. All animations featured two characters, a small blue triangle and a big red one, whom both moved on a white background. Stimuli were presented on a 17-inch display with a 60 Hz refresh rate and an 800 × 600 pixels resolution, viewed from 65 cm in a dimly lit room. Participants were instructed to observe the animation because they would be asked to describe what they had seen. Eye movements were recorded monocularly with a video-based desktop mounted eye tracker (see Supplementary Information 2 for a description of the eyetracking apparatus). After each animation, participants were asked to describe freely what they had seen. Their answers were recorded for off-line scoring by two raters blind to group membership.

#### Accuracy/intentionality scales

Answers were evaluated according to three dimensions using the usual scoring criteria provided by Castelli *et al*.^51^: intentionality (mentalistic complexity of the vocabulary used, 0–5 points), appropriateness (degree of correctness of descriptions, 0–2 points) and length scales (number of utterances, 1–4 points). The inter-rater correlation was high (mean weighted Cohen’s *kappa* across the 3 scales: 0.81) and the two raters’ scores were averaged.

#### Contingency/intentionality scale

The aforementioned intentionality scale mixes indices of contingency (whether the described actions imply a connection, an interaction or an influence between the two triangles) and intentionality (whether the described actions have been done on purpose). For example, the fourth step of the scale is “deliberate action in response to other’s action” which involves both contingency and intentionality. This might be a problem, as intentionality and contingency have been characterized as two separate dimensions in schizophrenia^22^,^23^. For this reason, we have designed a new scale aiming at disentangling these two dimensions in participant’s answers. The described actions for every animation were classified according to 4 categories: intentional contingent, intentional non-contingent, mechanical contingent and mechanical non-contingent (see Supplementary Information 3 for a complete description of the rating procedure). The four scores obtained for each trial showed high inter-rater correlation (mean weighted Cohen’s *kappa* across the 4 categories: 0.82) and the two raters’ scores were averaged.

### Eye movement measures

#### Fixation duration

After the exclusion of blinks, fixations were automatically detected with the EyeLink software algorithm (SR Research, Ontario, Canada) according to the following thresholds: eye velocity below 30°/sec, eye acceleration below 8000°/sec^2^ and eye movement amplitude smaller than 0.1°. This algorithm is designed to avoid short fixations (less than 100 ms in duration).

#### Triangle time

We considered that the gaze fell within a triangle if it fell within a circle whose center was the barycenter of the triangle and whose radius was the distance between the barycenter and the furthest corner of the triangle plus 1°. Triangle time was calculated as the cumulative duration of gaze within either the blue or the red triangle, divided by the total length of the animation.

### Statistical analyses

We first ran repeated-measures ANOVAs on accuracy, intentionality and length of verbal descriptions with Group as the between-subject factor and Condition as the within-subject factor. We then ran a repeated-measures ANOVA on the number of described actions with Group and as the between-subject factor, and Condition, Intentionality, and Contingency as the within-subject factors. We finally ran repeated-measures ANOVA on fixation durations and triangle time with Group as the between-subject factor and Condition as the within-subject factor. Results were corrected for sphericity using the Greenhouse-Geisser (GG) method where appropriate.

In a second step, we ran the same models as ANOVAs, adding covariates that might be confounding factors (such as IQ and cognitive control, but only if they were significantly associated with the dependent variable). This was done to test to what extent the results obtained might be due to such variables. The results of ANCOVAs are presented as Supplementary Information 5 and 10.

## Results

### Participants

Demographic, cognitive and clinical characteristics of the groups are shown in Table 1. Patients had a lower contextual control score, marginally lower IQ but were matched on age, sex, educational level.

### Verbal descriptions

#### Accuracy/intentionality scales

**Figure 1:**
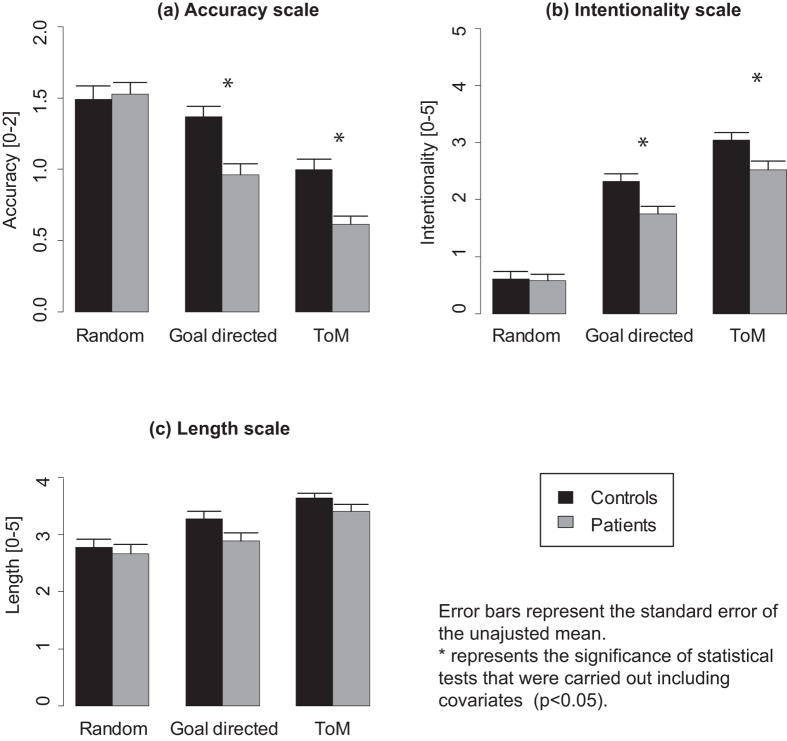
Mean (**a**) accuracy, (**b**) intentionality and (**c**) length of participants descriptions for random, goal-directed and theory of mind animations.

Three trials were excluded due to a lack of response (two for a patient, one for a control participant). Barplots are presented in Fig. 1 and boxplots in Supplementary Information 4.

The ANOVA run on the length scale revealed a significant effect of Condition (F(2, 112) = 69.7, p[GG] < 0.001) but no significant effects for Group (F(1, 56) = 2, p = 0.16) and Group by Condition interaction (F(2, 112) = 2, p[GG] = 0.14). Length was greater for GD than for R (F(1, 57) = 240, p < 0.001) and greater for ToM than for R (F(1, 57) = 363.6, p < 0.001) and GD (F(1, 57) = 58.9, p < 0.001).

The ANOVA run on the accuracy scale revealed a significant Group by Condition interaction (F(2, 112) = 6.8, p[GG] = 0.002). The post-hoc tests revealed that Group was significant for GD (F(1, 56) = 14.5, p < 0.001) and ToM (F(1, 56) = 16.7, p < 0.001) but not for R (F(1, 56) = 0.1, p = 0.76). Accuracy was lower for patients compared to controls for GD and ToM conditions. ANCOVAs (Supplementary Information 5) showed that contextual control and IQ did not explain group differences.

For controls, accuracy was not different between R and GD (F(1, 28) = 2.3, p = 0.15), greater in R than ToM (F(1, 28) = 23.5, p < 0.001) and greater in GD than ToM (F(1, 28) = 19.1, p < 0.001). For patients, accuracy was greater in R than in ToM (F(1, 28) = 113, p < 0.001) and GD (F(1, 28) = 20.6, p < 0.001) and greater in GD than in ToM (F(1, 28) = 16.1, p < 0.001).

The ANOVA run on the intentionality scale revealed a significant Group by Condition interaction (F(2, 112) = 4.5, p[GG] = 0.015). The post-hoc tests revealed that Group was significant for ToM (F(1, 56) = 6.4, p = 0.015), GD (F(1, 56) = 9.4, p = 0.003) but not for R (F(1, 56) = 0.04, p = 0.84). Figure 1b showed that intentionality scores were lower for patients compared to controls for GD. ANCOVAs (Supplementary Information 5) showed that contextual control and IQ did not explain group differences in intentionality for GD; however, contextual control explained most of the group difference in intentionality for ToM.

For controls, intentionality was greater for GD than for R (F(1, 28) = 169.2, p < 0.001) and greater for ToM than for R (F(1, 28) = 224.6, p < 0.001) and GD (F(1, 28) = 27.6, p < 0.001). For patients, intentionality was greater for GD than for R (F(1, 28) = 107.4, p < 0.001) and greater for ToM than for R (F(1, 28) = 164, p < 0.001) and GD (F(1, 28) = 30.4, p < 0.001).

#### Contingency/intentionality scale

**Figure 2:**
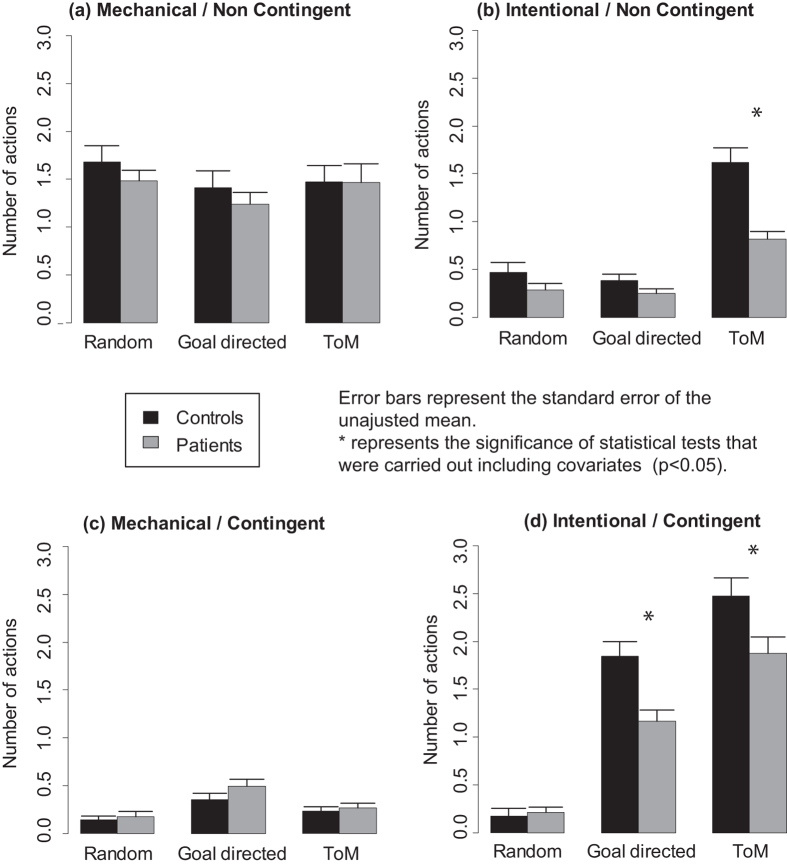
Results for the contingency/intentionality scale with mean number of (**a**) mechanical/non contingent, (**b**) intentional/non contingent, (**c**) mechanical/contingent and (**d**) intentional/contingent actions in participants’ descriptions for random, goal directed and theory of mind animations.

Barplots are presented in Fig. 2 and boxplots in Supplementary Information 6.

The ANOVA run on the contingency/intentionality scale revealed a significant triple interaction between Condition, Intentionality, and Group (F(2, 112) = 6.8, p[GG] = 0.002). This suggests that patients and controls differed in their descriptions of intentional actions, but not in the same way for R, GD and ToM animations. However, neither the triple interaction between Condition, Contingency, and Group (F(2, 112) = 1.9, p[GG] = 0.16) nor the interaction between Contingency and Group (F(1, 56) = 0.5, p = 0.47) were significant. This suggests that patients and controls did not differ in their descriptions of contingent actions. The quadruple interaction between Condition, Intentionality, Contingency and Group was significant (F(2, 112) = 4.9, p[GG] = 0.011).

The post-hoc tests revealed that Group was significant for intentional contingent actions in ToM (F(1, 56) = 5.4, p = 0.024) and GD (F(1, 56) = 12.1, p < 0.001) but not in R (F(1, 56) = 0.1, p = 0.74). Group was also significant for intentional non-contingent actions in ToM (F(1, 56) = 25.2, p < 0.001) but not in GD (F(1, 56) = 2.5, p = 0.12) and R (F(1, 56) = 2.3, p = 0.13). Group was not significant for mechanical non-contingent actions in ToM (F(1, 56) = 0, p = 0.97), GD (F(1, 56) = 0.6, p = 0.43) and R (F(1, 56) = 0.9, p = 0.34). Group was not significant for mechanical contingent actions in ToM (F(1, 56) = 0.2, p = 0.62), GD (F(1, 56) = 1.9, p = 0.17) and R (F(1, 56) = 0.3, p = 0.61). ANCOVAs (Supplementary Information 5) showed that contextual control and IQ did not explain group differences. The number of described actions was lower for patients compared to controls for intentional non-contingent actions in ToM and intentional contingent actions in GD. The post-hoc tests for the remaining contrasts are presented in Supplementary Information 7.

There was no significant correlation between verbal descriptions of Frith-Happé stimuli and clinical symptoms (see Supplementary information 8).

### Ocular measures

**Figure 3:**
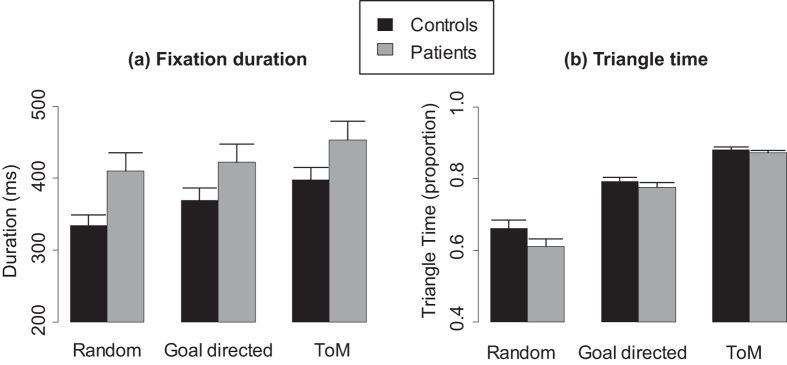
Mean (**a**) fixation duration, and (**b**) triangle time for random, goal-directed and theory of mind animations. Error bars represent the standard error of the mean.

Barplots are presented in Fig. 3 and boxplots in Supplementary Information 9.

There was no fixation whose duration was below 100 ms (the shortest fixation duration was 199.8 ms). The ANOVA run on fixation duration revealed significant effects of Group (F(1, 56) = 4.2, p = 0.045), Condition (F(2, 112) = 36.1, p[GG] < 0.001) but no significant interaction between Group and Condition (F(2, 112) = 2, p[GG] = 0.15). Fixation duration was lower for controls compared to patients. Fixation duration was greater for GD than for R (F(1, 57) = 15.8, p < 0.001) and greater for ToM than for R (F(1, 57) = 50.8, p < 0.001) and GD (F(1, 57) = 30.1, p < 0.001). Correlation analyses (Supplementary Information 10) showed that contextual control and IQ did not explain group differences for fixation duration.

The ANOVA run on triangle time revealed a significant effect of Condition (F(2, 112) = 234.7, p[GG] < 0.001) but no significant effects of Group (F(1, 56) = 2.2, p = 0.14) and Group by Condition interaction (F(2, 112) = 2, p = 0.15). Triangle time was greater for GD than for R (F(1, 57) = 189.4, p < 0.001) and greater for ToM than for R (F(1, 57) = 267.3, p < 0.001) and GD (F(1, 57) = 169.2, p < 0.001).

Finally, exploratory correlation analyses revealed no significant correlation between implicit mentalizing and clinical symptoms (see Supplementary Information 11), and no significant correlation between implicit and explicit mentalizing measures, except for controls in the GD condition (see Supplementary Information 12).

## Discussion

In this study, we used Frith-Happé animations to assess the ability to attribute intentions and contingency in schizophrenia. Explicit mentalizing ability was measured from participants’ verbal descriptions of the animations. Because little is known about how individuals with schizophrenia extract relevant cues when observing animated social agents, eye movements were recorded while participants were watching Frith-Happé animations. We examined whether participants with schizophrenia would show the same modulation of eye movements by the different types of animations as control participants, in the hope of obtaining some insight into implicit mentalizing processes.

### Explicit mentalizing

As in previous studies, individuals with schizophrenia differed from controls in the way they described the animations: they made less accurate and intentional description of GD and ToM animations. No group differences were found in the R condition, suggesting that this deficit was not just a general decrease in the ability to make verbal descriptions.

We found no evidence for hypermentalizing in schizophrenia, as patients did not attribute more intentions to triangles in any condition compared to controls. Neither did we find evidence for apophenia in schizophrenia, as patients did not attribute more contingency between the two moving triangles in any condition compared to controls. This last result is the opposite of the one reported by Blakemore *et al*. who found a normal attribution of intentions but an increased attribution of contingency in a transnosographical group of 22 deluded patients^23^. These results suggest that whereas delusion per se might be related to an overattribution of contingency, schizophrenia seems better characterized by a decreased attribution of intentions.

It is worth noting that group differences in explicit mentalizing were not explained by cognitive control, verbal and performance IQ. However, contextual processing was associated with the accuracy of verbal description (see Supplementary Information 5), consistently with other studies suggesting a partial overlap between executive dysfunction and impairment of social cognition in schizophrenia^39^,^52^. However, these results are inconsistent with hypotheses suggesting that social cognitive deficits in schizophrenia are entirely attributable to contextual processing deficits.

The study did not show significant correlations between clinical symptoms and explicit measures of mentalizing. However, social cognition usually correlates moderately with disorganization and negative symptoms of schizophrenia (with r ranging between −0.2 to −0.32)^53^: thus our study was not suitably powered to investigate correlations between symptoms and explicit mentalizing.

### Implicit mentalizing

The eyetracking results revealed that individuals with and without schizophrenia showed a similar modulation of eye movements in response to the different conditions of the Frith-Happé animations. First, both groups showed the same increase in fixation duration from R to ToM animations, consistently with previous studies^42^,^46^,^47^,^48^. This suggests an equal increase in cognitive processing related to the integration of mental states in patients as in controls. An increase in fixation duration regardless of the type of animation was found in patients. This is consistent with early studies suggesting that schizophrenia has been consistently associated with an increase in average fixation durations for a broad range of visual stimuli in different tasks, as well as with fewer fixations and saccades, smaller saccades and shorter scanpath length than controls^54^. This increase has been related to difficulties in attentional disengagement, the speed of information processing and a restricted visual scanning strategy in schizophrenia.

Gaze was spontaneously directed to the intentional triangles (GD and TOM conditions) for longer durations than to the random ones, thus replicating the modulation of triangle time by the type of animation found in others studies^46^,^47^,^48^. Triangle time, an indicator of how much eye movements are preferentially directed to the intentional triangles, was also similar in both groups, thus suggesting that the detection and early processing of goal-directed actions and complex mental states are spared in schizophrenia. Overall, the eye tracking results indicate that the implicit and early stages of mentalizing are preserved in schizophrenia. Of course, eyetracking can only tell us what information has been made available to the participants, but cannot reveal exactly to what extent that information has been processed. However, the fact that patients’ triangle time was modulated to the same extent as controls’ by the experimental manipulation of mental state content and complexity does suggest that they processed enough relevant information to distinguish the experimental conditions, hence that at least early stages of mentalizing were preserved. Only later stages of mentalizing, including the ability to report an intentional and fitting description of intention and mental states, were found to be affected in schizophrenia in this study. These results are consistent with Frith’s hypothesis of a dissociation between preserved implicit, spontaneous mentalizing and abnormal explicit mentalizing in schizophrenia^9^. Interestingly, a dissociation between explicit and implicit mentalizing has also been suggested for autism, but implicit ToM has been suggested to be impaired, whereas explicit ToM seems to be preserved thanks to the development of secondary compensatory learning strategies^55^,^56^.

One limitation to the present study came from the fact that the paradigm was not entirely implicit, as participants were required to focus their attention on the animations because they would be asked to describe them. In an entirely implicit design, participants would have been asked to perform a task unrelated to the description of animations, while eye movements were recorded. This study does not explore the stages between the visual processing of triangles and the interpretation of the scene. Another limitation was related to the DSM-IV-R diagnosis interviews of patients and controls that did not involve a structured instrument like the Structured Clinical Interview for Disorders. There was a discrepancy between the results on the length scale and the results on the contingency/intentionality scale. Although descriptions were as long in patients and controls, patients described fewer intentional actions in GD and ToM animations than controls. This decrease was not compensated by an increase in the description of the mechanical movements in these two conditions. There was a global reduction in the number of actions described in these two conditions: patients might have committed more repetitions, or described the triangles more on their physical appearance rather on their actions. The present results could thus also be interpreted according to the experiential (perceptual) vs. narrative dichotomy, as patients were impaired in the verbal description of GD and ToM animations. Indeed, schizophrenia has been characterized by an uninformative speech with a low idea density^57^: our results suggest that this deficit seems to occur only when they have to infer simple and complex intentions, but not mechanical actions. Finally, the paradigm used two different and heterogeneous conditions of mentalizing. The first condition (GD) implies a basic form of mentalizing, with the attribution of simple intentions to others. The second condition (ToM) involves a complex form of mentalizing, with the attribution of complex intentions to others. It is worth noting that differences in intentionality between patients and controls were partly explained by contextual control difference for ToM but not for GD: complex mentalizing may require complex cognitive processing like the integration of different sources of contextual information whereas basic mentalizing may rely more on perceptual information. The final limitation relates to the impact of the kinematic differences on the interpretation of eye tracking differences between GD and ToM. In a previous study, we had demonstrated that kinematic confounds did not explain eye movement differences between R and GD and between R and ToM. However, eye movement differences between GD and ToM were entirely explained by kinematic confounds. Thus our study validated the dissociation between explicit mentalizing and implicit attribution of intentions in general: however, it was not possible to compare the basic implicit mentalizing with the complex explicit one.

To conclude, we found that patients with schizophrenia attributed fewer mental states to the Frith-Happé triangles but had a normal attribution of contingency between these two agents. This deficit in their ability to explicitly mentalize was dissociated from a preserved implicit mentalizing, as revealed by their eye movements.

## Additional Information

**How to cite this article**: Roux, P. *et al*. Preserved implicit mentalizing in schizophrenia despite poor explicit performance: evidence from eye tracking. *Sci. Rep.* **6**, 34728; doi: 10.1038/srep34728 (2016).

## Supplementary Material

Supplementary Information

## Figures and Tables

**Table 1:** Characteristics of participants.

Variable	Schizophrenic participants		Controls		Statistics	*p* value
N	29		29			
Sex ratio (M/F)^1^	21/8		19/10		*Χ^2^* = 0.1	0.777
Visual correction (CL/G)^2^	1/12		3/9		*Χ^2^* = 0^3^	1
	**Mean**	**SD**	**Mean**	**SD**		
Age (years)	39	12.5	40.7	13.5	t(56) = 0.5	0.63
Educational level (years)	12	2.3	12.4	1.5	t(56) = 0.9	0.389
Estimated General Intelligence^4^	8.3	2.1	9.3	2.1	t(56) = 1.8	0.08
Contextual control (error rate)^5^	0.17	0.13	0.09	0.1	F(1, 55) = 6.3	**0.015**
Illness duration (years)	18	11.1				
Hospitalizations duration (months)	16.5	19.3				
Haloperidol equivalents (mg/24 h)	11.7	8.6				
PANSS total	90.6	12				
PANSS Positive factor	20.7	3.5				
PANSS Negative factor	24.1	4.5				
PANSS Disorganization factor	20.2	3.8				
PANSS Excitement factor	14.6	2.5				
PANSS Anxiety/Depression factor	8.2	2.9				

^1^male/female.

^2^contact lenses/glasses.

^3^For the chi-square test, contact lenses and glasses were counted as one category due to small sample size.

^4^Mean scaled scores, from 1 to 19. Wechsler intelligence scaled scores have a mean of 10 and a standard deviation of 3 in the general population.

^5^For the high contextual control condition only.
