# Supplementary material for: Preserved implicit mentalizing in schizophrenia despite poor explicit performance: evidence from eye tracking
Source: Sci Rep. 2016 Oct 5;6:34728. doi: 10.1038/srep34728 (PMC5050453; doi:10.1038/srep34728)
Supplement: Supplementary Information [file srep34728-s1.doc]

**Preserved implicit mentalization in schizophrenia despite poor explicit performance: evidence from eye tracking**

**Supplementary Information**

Paul Roux, Pauline Smith, Christine Passerieux, Franck Ramus

# Supplementary Information 1. The contextual cognitive control experiment

Participants were presented a series of colored letters and had to respond by pressing two response buttons. Each participant underwent four blocks including 16 sequences of 12 stimuli (duration: 500 ms; onset asynchrony: 3500 ms) preceded by an instruction. In contrast with the original paradigm, participants controlled the duration of instruction presentation to ensure that they had enough time to read and understand the instructions. Participants performed two tasks according to the color of the letter: a lower/upper case discrimination task (T1) when the letter was green, a consonant/vowel discrimination task (T2) when the letter was red. In each task, participants had to ignore the stimulus when it was white. In low contextual control sequences, participants were presented with only red/white or only green/white letters and performed only one task during a given sequence. In high contextual control sequences, participants were presented with a mixture of red, green and white letters and had to select between T1 and T2 according to the color of the letter.

Supplementary Figure S1. Schematic representation of the contextual cognitive control paradigm

Stimuli were pseudo-randomized according to several rules: two successive stimuli were never identical, no more than three consecutive identical responses, the ratio of left, right and no responses and the proportion of congruent versus incongruent letters (same versus different responses for T1 and T2) were equal to 1. Participants were trained on low (one sequence of T1, one sequence of T2) and high contextual control sequences (two T1/T2 sequences). Participants had to obtain a minimal threshold of 2/3 correct responses on every sequence. Otherwise, the sequence went on until the threshold was reached.

As demonstrated in the original study ^1^, it was hypothesized that patients with schizophrenia were selectively impaired in the high contextual control condition but not in the low contextual control condition.

A repeated measures analysis of variance (ANOVA) was run on error rate with one between-subject factor, Group (two modalities: patient or control) and one within-subject factor, Contextual Demand (two modalities: high and low contextual control demand).

One patient could not perform the task because she did not reach the criterion in the high contextual control sequences during the training phase, whereas she managed to reach this criterion for T1 and T2 low contextual control sequences. The missing measure for this participant was replaced by the highest error rate in the high control condition obtained in the group of patients (37.5 %). This replacement avoids excluding a patient with low contextual control ability who was able to perform all other tasks.

Results are presented in Supplementary Figure S2

.

Supplementary Figure S2. Mean error rates for the low and high contextual control conditions. Error bars represent the standard error.

The analysis showed a significant effect of Group (F(1,55)=6.3, **p=0.015**), a significant effect of Contextual Demand (F(1,56)=59.4, **p<10^-3^** ) but no interaction between Group and Contextual Demand (F(1,56)=0.8, p=0.375). Figure 2 illustrates that patients were impaired both for low and high contextual control conditions compared to controls.

To conclude, patients were equally impaired in low and high cognitive control conditions, which was not expected because the original study ^1^ found that patients with schizophrenia were selectively impaired in the high contextual control condition. This discrepancy might be explained by the fact that participants were much more trained to the general procedure of this task in the original study compared to the present study, which may have helped them achieve normal performance in the easier condition.

However, we replicated that patients were impaired in the high contextual control condition. In this study, we were mainly interested in a measure of contextual control in schizophrenia to check whether it could influence spontaneous mentalizing in schizophrenia. We selected the error rate in the high control condition as the measure of contextual control.

# Supplementary Information 2. Eyetracking apparatus

The experiments were run with Matlab using the Psychophysics and Eyelink Toolboxes ^2,3^. Eye movements were recorded with Eyelink 1000 system (Remote/Head Free configuration, SR Research, Ontario, Canada), a sampling rate of 500 Hz and a spatial resolution of 1°. Before the beginning of the experiment, the eye tracker was set to obtain the best pupil and corneal reflection images for each participant. A 9-dot calibration procedure was done before the three training trials and before the 1st, 5th, and 9th test animations. Before each trial, a “drift correct” marker was presented in the center of the screen. Participants were required to look at the dot and press a response button when fixation was attained. This constrained the initial position of fixation and triggered a new calibration if the eye drift was greater than 5°. Participants were instructed to avoid blinking as much as possible during each animation.

# Supplementary Information 3. The intentionality/contingency scale

This scale was inspired from a method developed for Frith-Happé animations that computed two scores ^4^ :

- an apophenia score (overattribution of contingencies) obtained by summing the number of connections in responses to random items
- an overmentalizing score (overattribution of mental states) obtained by summing the number of references made to the thoughts, feelings, beliefs, intentions, goal-directed or interactive behaviors in responses to random items

However, this method does not control for covariation between apophenia and hypermentalizing. It also restricts the evaluation of contingency and intentionality to the random condition only. In the present study, described actions for every animation were classified according to 4 categories:

1. **intentional contingent**. The action is clearly deliberate, and triangles are interacting with each other (i.e. "they dance with each other", "one triangle is chasing the other one")
2. **intentional non-contingent**. The action is clearly deliberate but does not imply a relation between the two triangles (i.e. "the triangles are walking around", "a triangle is wondering something")
3. **mechanical contingent**. The action is non-deliberate but implies a relation between the two triangles (i.e. "they are pulled by each other like a magnet", "their trajectories are symmetrical")
4. **mechanical non-contingent**. The action is neither deliberate nor interactive ("the triangles are moving", "they appear on the left side of the screen")

Thus, four scores were obtained for each trial. There was no upper limit to the number of described actions. This was done because animations are quite complex (particularly in ToM) and long (40 seconds), such that an accurate description of these animations requires describing several actions. We did not count repetition in the description of the same action. Allowing the count of multiple actions thus gives a quantitative insight into the richness of contingency and intention attribution. When no action was described (e.g. “there is a blue and a red triangle”), the score was zero.

This two by two Latin square design allowed us to explore the respective effects of intentionality, contingency and their interaction on the number of described actions.

# Supplementary Information 4. Boxplots of the accuracy, intentionality, and length scales.

## Length scale

## Accuracy scale

## Intentionality scale

# Supplementary Information 5. The impact of Verbal IQ, Performance IQ and Contextual Control on the verbal descriptions of Frith-Happé stimuli.

## Accuracy scale

Accuracy correlated significantly with Contextual Control (t(56)=2.5, **p=0.015**, Pearson's r=0.32), and Performance (t(56)=4.2, **p<0.001**, Pearson's r=0.49) but not with Verbal IQ (t(56)=0.44, p=0.66, Pearson's r=0.06). In order to check whether neurocognitive confounds might explain group differences in mentalization, we have run a repeated-measures analysis of covariance on accuracy with Group as the between-subject factor, Condition as the within-subject factor and Performance IQ and Contextual Control as covariates. It revealed a significant Group by Condition interaction (F(2,112)=6.8, **p[GG]=0.002**). The posthoc tests revealed that Group was significant for GD (F(1,56)=5.2, **p=0.027**) and ToM (F(1,56)=7.9, **p=0.007**) but not for R (F(1,56)=1.1, p=0.30).

## Intentionality scale

Intentionality correlated marginally with Contextual Control (t(56)=1.9, p=0.056, Pearson's r=0.25), but not with Performance (t(56)=0.2, p=00.81, Pearson's r=0.03) or 0Verbal IQ (t(56)=1.3, p=0.18, Pearson's r=0.18). In order to check whether group differences in mentalization might be explained by neurocognitive confounds, we have run a repeated-measures analysis of covariance on intentionality with Group as the between-subject factor, Condition as the within-subject factor and Contextual Control as the covariate. It revealed a significant Group by Condition interaction (F(2,112)=4.5, **p[GG]=0.01**0**5**). The post-hoc tests revealed that Group was significant for GD (F(1,56)=5.9, **p=0.018**), marginally significant for ToM (F(1,56)=2.9, p=0.092) but not for R (F(1,56)=0.1, p=0.78).

## Contingency / intentionality scale

The number of described actions correlated significantly with Verbal IQ (t(56)=2, **p=0.047**, Pearson's r=0.26), but not with Performance IQ (t(56)=1.3, p=00.19, Pearson's r=0.17) or 0 Contextual Control (t(56)=0.9, p=0.34, Pearson's r=0.13). To check whether neurocognitive confounds might explain group differences, we have run a repeated-measures analysis of covariance on the number of described actions with Group as the between-subject factor, Condition, Contingency and Intentionality as the within-subject factor and Verbal IQ as the covariate. It revealed a significant quadruple interaction between Condition, Intentionality, Contingency and Group (F(2,112)=4.9, **p[GG]=0.011**).

The post-hoc tests revealed that Group was significant for intentional contingent actions in ToM (F(1,56)=4.7, **p=0.035**) and GD (F(1,56)=11.2, **p=0.001**) but not in R (F(1,56)=0.4, p=0.55). Group was also significant for intentional non contingent actions in ToM (F(1,56)=19.5, **p<0.001**) but not in GD (F(1,56)=2.1, p=0.15) and R (F(1,56)=2.3, p=0.13). Group was not significant for mechanical non contingent actions in ToM (F(1,56)=0, p=0.97), GD (F(1,56)=0.6, p=0.44) and R (F(1,56)=0.8, p=0.37). Group was not significant for mechanical contingent actions in ToM (F(1,56)=0.2, p=0.66), GD (F(1,56)=1.8, p=0.18) and R (F(1,56)=0.3, p=0.60).

# Supplementary Information 6. Boxplots of the contingency/intentionality scale.

## Mechanical non-contingent actions

## Intentional non-contingent actions

## Mechanical contingent actions

## Intentional contingent actions

# Supplementary Information 7. Remaining posthoc tests for the ANOVA run on the number of described actions with Group and as the between-subject factor, and Condition, Intentionality, and Contingency as the within-subject factors.

## Contrasting the type of animations

For controls, the number of intentional contingent actions was greater for GD than for R (F(1,28)=121.5, p<0.001) and greater for ToM than for R (F(1,28)= 169.4, p<0.001) and GD (F(1,28)= 11.8, p=0.002). For patients, the number of intentional contingent actions was greater for GD than for R (F(1,28)=77, p<0.001) and greater for ToM than for R (F(1,28)= 107, p<0.001) and GD (F(1,28)= 18.6, p= p<0.001).

For controls, the number of intentional non contingent actions was not different between GD and R (F(1,28)=0.7, p=0.40) but was greater for ToM than for R (F(1,28)= 38.9, p<0.001) and GD (F(1,28)= 56.3, p<0.001). For patients, the number of intentional non contingent actions was not different between GD and R (F(1,28)=0.3, p=0.57) but was greater for ToM than for R (F(1,28)= 24.8, p<0.001) and GD (F(1,28)= 37.8, p<0.001).

For controls, the number of mechanical non contingent actions was smaller for GD than for R (F(1,28)=4.5, p=0.044) but not different between ToM and R (F(1,28)= 2.5, p=0.12) and between ToM and GD (F(1,28)= 0.3, p=0.60). For patients, the number of mechanical non contingent actions was smaller for GD than for R (F(1,28)=5.5, p=0.033) but not different between ToM and R (F(1,28)= 0.02, p=0.89) and between ToM and GD (F(1,28)= 2.3, p=0.14).

For controls, the number of mechanical contingent actions was greater for GD than for R (F(1,28)=11.7, p=0.002), marginally greater for ToM than for R (F(1,28)= 3.3, p=0.08) and marginally smaller for ToM than for GD (F(1,28)= 4, p=0.055). For patients, the number of mechanical contingent actions was greater for GD than for R (F(1,28)=11.2, p=0.002), not different between ToM and R (F(1,28)= 2.2, p=0.15) and significantly smaller for ToM than for GD (F(1,28)= 8.1, p=0.008).

## Contrasting intentionality

For controls, the number of mechanical non contingent actions was greater than the number of intentional non contingent actions in R (F(1,28)=27.6, p<0.001) and GD (F(1,28)=28.5, p<0.001). For controls, the number of mechanical non contingent actions was not different from the number of intentional non contingent actions in ToM (F(1,28)=0.4, p=0.54). For patients, the number of mechanical non contingent actions was greater than the number of intentional non contingent actions in R (F(1,28)=65.8, p<0.001), GD (F(1,28)=46.2, p<0.001) and ToM F(1,28)=12.7, p=0.001).

For controls, the number of mechanical contingent actions was smaller than the number of intentional contingent actions in GD (F(1,28)=85.8, p<0.001) and ToM (F(1,28)=147.4, p<0.001). For controls, the number of mechanical contingent actions was not different from the number of intentional contingent actions in R (F(1,28)=0.1, p=0.70). For patients, the number of mechanical contingent actions was smaller than the number of intentional contingent actions in GD (F(1,28)=24.1, p<0.001) and ToM (F(1,28)=75, p<0.001). For patients, the number of mechanical contingent actions was not different from the number of intentional contingent actions in R (F(1,28)=0.3, p=0.57).

## Contrasting contingency

For controls, the number of mechanical non contingent actions was greater than the number of mechanical contingent actions in R (F(1,28)=92, p<0.001), GD (F(1,28)=11, p<0.001) and ToM (F(1,28)=69, p<0.001). For controls, the number of mechanical non contingent actions was greater than the number of mechanical contingent actions in R (F(1,28)=112, p<0.001), GD (F(1,28)=34.4, p<0.001) and ToM (F(1,28)=42.8, p<0.001).

For controls, the number of intentional non-contingent actions was smaller than the number of intentional contingent actions in GD (F(1,28)=97.7, p<0.001) and ToM (F(1,28)=21.2, p<0.001). For controls, the number of intentional non-contingent actions was greater than the number of intentional contingent actions in R (F(1,28)=9, p=0.006). For patients, the number of intentional non-contingent actions was smaller than the number of intentional contingent actions in GD (F(1,28)=60.7, p<0.001) and ToM (F(1,28)=34.8, p<0.001). For patients, the number of intentional non-contingent actions was not different from the number of intentional contingent actions in R (F(1,28)=0.9, p=0.36).

# Supplementary Information 8. Spearman's correlations between verbal descriptions of Frith-Happé animations and clinical symptoms.

## Accuracy scale

|  | Accuracy for R | | Accuracy for GD | | Accuracy for ToM | |
| --- | --- | --- | --- | --- | --- | --- |
| **Positive and Negative Syndrome Scale** | ρ | P | ρ | P | ρ | P |
| Positive | -0.14 | 0.469 | 0.14 | 0.484 | 0.28 | 0.142 |
| Negative | -0.14 | 0.479 | -0.15 | 0.43 | -0.25 | 0.195 |
| Disorganization | -0.22 | 0.243 | -0.24 | 0.218 | -0.33 | 0.084 |
| Excitement | -0.18 | 0.35 | -0.27 | 0.153 | 0.05 | 0.796 |
| Emotional distress | 0.15 | 0.432 | -0.16 | 0.401 | -0.14 | 0.467 |

Spearman's correlations between the scores on accuracy scale and clinical symptoms according to the 5-factors model of van der Gaag et al. ^5^.

The alpha-level was set to 0.05/15=0.003, as a correction for multiple comparisons with the Bonferroni method. There was no significant correlation between accuracy scale and symptoms measured with the PANSS.

## Intentionality scale

|  | Intentionality for R | | Intentionality for GD | | Intentionality for ToM | |
| --- | --- | --- | --- | --- | --- | --- |
| **Positive and Negative Syndrome Scale** | ρ | P | ρ | P | ρ | P |
| Positive | 0.18 | 0.34 | 0.15 | 0.44 | 0.27 | 0.16 |
| Negative | 0.14 | 0.46 | -0.32 | 0.09 | -0.35 | 0.066 |
| Disorganization | 0.21 | 0.282 | -0.06 | 0.771 | -0.18 | 0.357 |
| Excitement | 0.09 | 0.639 | -0.13 | 0.494 | -0.1 | 0.601 |
| Emotional distress | -0.11 | 0.586 | -0.28 | 0.145 | -0.21 | 0.278 |

Spearman's correlations between the scores on intentionality scale and clinical symptoms according to the 5-factors model of van der Gaag et al. ^5^.

The alpha-level was set to 0.05/15=0.003, as a correction for multiple comparisons with the Bonferroni method. There was no significant correlation between accuracy scale and symptoms measured with the PANSS.

## Contingency / intentionality scale

In these correlation analyses, we have explored whether the attribution of intention and contingency were related with clinical symptoms. We thus have used only two measures of the contingency/intentionality scale: the number of intentional actions and the number of contingent actions. These two measures are consistent with the two main dimensions of the contingency/intentionality scale. We have not used the four measures obtained by the combination of these two dimensions (intentional contingent, intentional non-contingent, mechanical contingent and mechanical non-contingent).

|  | Number of intentional actions for R | | Number of intentional actions for GD | | Number of intentional actions for ToM | |
| --- | --- | --- | --- | --- | --- | --- |
| **Positive and Negative Syndrome Scale** | ρ | P | ρ | P | ρ | P |
| Positive | 0.3 | 0.11 | 0.26 | 0.181 | 0.39 | 0.037 |
| Negative | 0.26 | 0.169 | -0.21 | 0.274 | 0.06 | 0.773 |
| Disorganization | 0.14 | 0.459 | 0 | 0.991 | 0.09 | 0.65 |
| Excitement | 0.13 | 0.488 | 0.04 | 0.825 | -0.1 | 0.607 |
| Emotional distress | 0.22 | 0.255 | -0.25 | 0.195 | -0.03 | 0.866 |

Spearman's correlations between the intentionality score on the contingency-intentionality scale and clinical symptoms according to the 5-factors model of van der Gaag et al. ^5^.

The alpha-level was set to 0.05/15=0.003, as a correction for multiple comparisons with the Bonferroni method. There was no significant correlation between the intentionality score on the contingency-intentionality scale and symptoms measured with the PANSS.

|  | Number of contingent actions for R | | Number of contingent actions for GD | | Number of contingent actions for ToM | |
| --- | --- | --- | --- | --- | --- | --- |
| **Positive and Negative Syndrome Scale** | ρ | P | ρ | P | ρ | P |
| Positive | 0.06 | 0.767 | 0.07 | 0.719 | 0.43 | 0.02 |
| Negative | 0.18 | 0.339 | -0.18 | 0.342 | 0.21 | 0.28 |
| Disorganization | 0.17 | 0.372 | -0.1 | 0.595 | 0.37 | 0.046 |
| Excitement | -0.12 | 0.538 | 0.05 | 0.809 | 0.09 | 0.627 |
| Emotional distress | 0.27 | 0.155 | -0.32 | 0.093 | -0.06 | 0.749 |

Spearman's correlations between the contingency score on the contingency-intentionality scale and clinical symptoms according to the 5-factors model of van der Gaag et al. ^5^.

The alpha-level was set to 0.05/15=0.003, as a correction for multiple comparisons with the Bonferroni method. There was no significant correlation between the contingency score on the contingency-intentionality scale and symptoms measured with the PANSS.

# Supplementary Information 9. Boxplots of ocular measures.

## Fixation duration (ms)

## Triangle time (ms)

# Supplementary Information 10. Impact of Verbal IQ, Performance IQ and Contextual Control on the ocular measures.

Fixation duration did not correlated significantly with Contextual Control (t(56)=0.1, p=0.94, Pearson's r=0.01), Performance (t(56)=0.6, p=0.53**,** Pearson's r=0.08) and Verbal IQ (t(56)=0.3, p=0.80, Pearson's r=0.03).

# Supplementary Information 11. Spearman's correlations between ocular measures on Frith-Happé animations and clinical symptoms.

|  | Fixations for R | | Fixations for GD | | Fixations for ToM | |
| --- | --- | --- | --- | --- | --- | --- |
| **Positive and Negative Syndrome Scale** | ρ | P | ρ | P | ρ | P |
| Positive | -0.22 | 0.254 | -0.05 | 0.785 | -0.2 | 0.302 |
| Negative | -0.08 | 0.68 | -0.07 | 0.702 | -0.07 | 0.723 |
| Disorganization | -0.35 | 0.066 | -0.36 | 0.057 | -0.33 | 0.082 |
| Excitement | -0.21 | 0.264 | -0.11 | 0.572 | -0.12 | 0.529 |
| Emotional distress | 0.06 | 0.763 | 0.01 | 0.954 | -0.04 | 0.83 |

Spearman's correlations between fixation duration and clinical symptoms according to the 5-factors model of van der Gaag et al. ^5^.

The alpha-level was set to 0.05/15=0.003, as a correction for multiple comparisons with the Bonferroni method. There was no significant correlation between fixation duration and symptoms measured with the PANSS.

Spearman's correlations between triangle time and clinical symptoms according to the 5-factors model of van der Gaag et al. ^5^.

|  | Triangle Time for R | | Triangle Time for GD | | Triangle Time for ToM | |
| --- | --- | --- | --- | --- | --- | --- |
| **Positive and Negative Syndrome Scale** | ρ | P | ρ | P | ρ | P |
| Positive | 0.11 | 0.58 | 0.25 | 0.193 | -0.03 | 0.892 |
| Negative | -0.07 | 0.702 | -0.05 | 0.783 | -0.07 | 0.7 |
| Disorganization | 0.21 | 0.284 | 0.39 | 0.034 | 0.08 | 0.683 |
| Excitement | 0.08 | 0.684 | 0.09 | 0.661 | 0.3 | 0.118 |
| Emotional distress | -0.07 | 0.704 | 0.04 | 0.854 | -0.04 | 0.853 |

The alpha-level was set to 0.05/15=0.003, as a correction for multiple comparisons with the Bonferroni method. There was no significant correlation between triangle time and symptoms measured with the PANSS.

# Supplementary Information 12. Correlation between implicit and explicit measures of mentalization

We ran Spearman's rank correlations between explicit and implicit mentalizing in controls and patients separately. The alpha-level was set to 0.05/12=0.004, as a correction for multiple comparisons with the Bonferroni method.

Results are presented in Supplementary Table S1 and S2.

|  |  | **Explicit measures of mentalizing** | | | | | |
| --- | --- | --- | --- | --- | --- | --- | --- |
|  |  | Accuracy scale | | | Intentionality scale | | |
|  |  | R | GD | ToM | R | GD | ToM |
| **Implicit measures of mentalizing** | Mean fixation duration | 0.38 | 0.14 | 0.32 | -0.40 | -0.39 | -0.08 |
|  | Triangle Time | -0.18 | **0.54*** | 0.36 | 0.18 | 0.01 | 0.20 |

*: p-value<0.004 (Bonferroni corrected alpha-level)

**Supplementary Table S1.** Spearman's correlation coefficients between explicit and implicit measures of mentalizing in controls and the significance of the associated statistical tests

|  |  | **Explicit measures of mentalizing** | | | | | |
| --- | --- | --- | --- | --- | --- | --- | --- |
|  |  | Accuracy scale | | | Intentionality scale | | |
|  |  | R | GD | ToM | R | GD | ToM |
| **Implicit measures of mentalizing** | Mean fixation duration | 0.11 | 0.04 | 0.11 | -0.16 | -0.1 | 0.13 |
|  | Triangle Time | -0.09 | 0.17 | 0.08 | 0.16 | 0.23 | -0.04 |

*: p-value<0.004 (Bonferroni corrected alpha-level)

**Supplementary Table S2.** Spearman's correlation coefficients between explicit and implicit measures of mentalizing in patients and the significance of the associated statistical tests

There was a significant correlation between accuracy rating and Triangle Time in the control group for GD (S=1848.7 p=0.002, ρ =0.55). The longer participants fixated the GD triangles, and the more accurate descriptions they produced of the corresponding animations. In contrast, this correlation between accuracy rating and Triangle Time for GD was not significant in patients (S= 3747.8, p=0.388, ρ =0.17). Accuracy rating and Triangle Time were not significantly correlated for ToM and R, in controls and patients. Overall these results do not reveal strong and consistent relationships between implicit and explicit measures of mentalizing.

# References for the Supplementary information

1 Chambon, V. et al. The architecture of cognitive control in schizophrenia. Brain 131, 962-970 (2008).

2 Brainard, D. H. The psychophysics toolbox. Spatial vision 10, 433-436 (1997).

3 Pelli, D. G. The VideoToolbox software for visual psychophysics: Transforming numbers into movies. Spatial vision 10, 437-442 (1997).

4 Fyfe, S., Williams, C., Mason, O. J. & Pickup, G. J. Apophenia, theory of mind and schizotypy: perceiving meaning and intentionality in randomness. Cortex 44, 1316-1325 (2008).

5 van der Gaag, M. et al. The five-factor model of the Positive and Negative Syndrome Scale II: a ten-fold cross-validation of a revised model. Schizophr Res 85, 280-287 (2006).
